# Oscillometry in Stable Single and Double Lung Allograft Recipients Transplanted for Interstitial Lung Disease: Results of a Multi-Center Australian Study

**DOI:** 10.3389/ti.2023.11758

**Published:** 2023-12-05

**Authors:** Joan P. Y. Sim, Kristopher Nilsen, Brigitte M. Borg, Bronwyn Levvey, Jaideep Vazirani, Samantha Ennis, Marshall Plit, Gregory I. Snell, David R. Darley, Katrina O. Tonga

**Affiliations:** ^1^ Lung Transplant and Thoracic Medicine Unit, St Vincent’s Hospital, Sydney, NSW, Australia; ^2^ St Vincent’s Hospital Clinical Campus, Faculty of Medicine and Health, The University of New South Wales, Sydney, NSW, Australia; ^3^ Lung Transplant and Respiratory Medicine Service, The Alfred Hospital, Melbourne, VIC, Australia; ^4^ School of Public Health and Preventive Medicine, Monash University, Melbourne, VIC, Australia; ^5^ Faculty of Medicine, Nursing and Health Science, Monash University, Melbourne, VIC, Australia; ^6^ Northern Clinical School, Faculty of Medicine and Health, The University of Sydney, Sydney, NSW, Australia

**Keywords:** interstitial lung disease, resistance, oscillometry, single and double lung transplantation, reactance

## Abstract

Peak spirometry after single lung transplantation (SLTx) for interstitial lung disease (ILD) is lower than after double lung transplantation (DLTx), however the pathophysiologic mechanisms are unclear. We aim to assess respiratory mechanics in SLTx and DLTx for ILD using oscillometry. Spirometry and oscillometry (tremoflo^®^ C-100) were performed in stable SLTx and DLTx recipients in a multi-center study. Resistance (R_5_, R_5–19_) and reactance (X_5_) were compared between LTx recipient groups, matched by age and gender. A model of respiratory impedance using ILD and DLTx data was performed. In total, 45 stable LTx recipients were recruited (SLTx *n* = 23, DLTx *n* = 22; males: 87.0% vs. 77.3%; median age 63.0 vs. 63.0 years). Spirometry was significantly lower after SLTx compared with DLTx: %-predicted mean (SD) FEV_1_ [70.0 (14.5) vs. 93.5 (26.0)%]; FVC [70.5 (16.8) vs. 90.7 (12.8)%], *p < 0.01*. R_5_ and R_5–19_ were similar between groups (*p = 0.94* and *p = 0.11*, respectively) yet X_5_ was significantly worse after SLTx: median (IQR) X_5_ [−1.88 (−2.89 to −1.39) vs. −1.22 (−1.87 to −0.86)] cmH_2_O.s/L], *p < 0.01*. R_5_ and X_5_ measurements from the model were congruent with measurements in SLTx recipients. The similarities in resistance, yet differences in spirometry and reactance between both transplant groups suggest the important contribution of elastic properties to the pathophysiology. Oscillometry may provide further insight into the physiological changes occurring post-LTx.

## Graphical Abstract

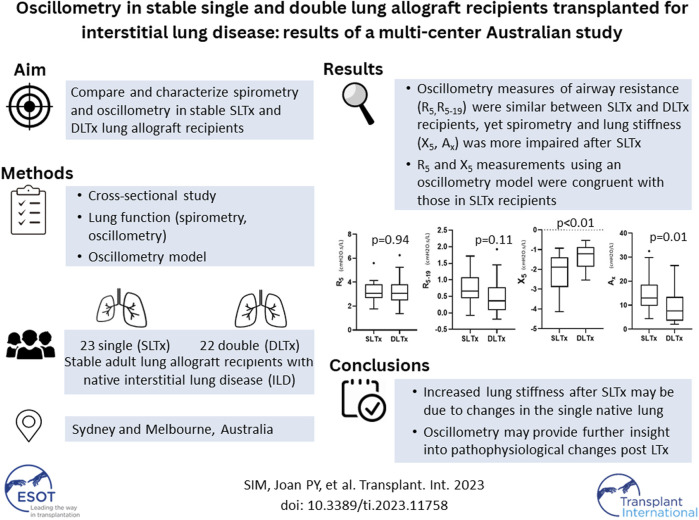

## Introduction

Lung transplantation (LTx) is an established intervention for patients with advanced interstitial lung disease (ILD) refractory to medical therapy [1]. LTx improves survival in patients with ILD [2] and outcomes depend on donor and recipient factors, choice of procedure and post-operative progress [3]. Single lung transplantation (SLTx) has been the predominant procedure used in patients with ILD, however, double lung transplantation (DLTx) is increasingly used [4]. Survival after LTx is limited by acute and chronic allograft dysfunction and subsequent failure, however there is conflicting data comparing outcomes post-SLTx versus DLTx [1,5,6].

Chronic allograft dysfunction is usually detected on spirometric surveillance [7] and defined as a persistent decline in the forced expiratory volume in one second (FEV_1_), from the best achieved post-operative FEV_1_ [8]. Studies have consistently demonstrated that FEV_1_ and forced vital capacity (FVC) are significantly lower in patients post-SLTx compared to post-DLTx during both short- and long-term follow up [9–11]. Lower spirometry post-SLTx may be attributed to disease progression in the contralateral native lung [9]. However, spirometry alone provides limited insight into the mechanisms contributing to the complex physiological differences between SLTx and DLTx. Furthermore, spirometry may be confounded and therefore produce variable results in SLTx recipients due to possible allograft compression during the forced breathing maneuver [12].

Oscillometry is a non-invasive lung function test performed during quiet tidal breathing that measures the respiratory mechanics of the chest wall, lung and airways [13]. During oscillometry measurement, pressure oscillations, usually of frequencies between 5 and 19 Hertz (Hz), are superimposed at the mouth [14]. The measured pressure and airflow changes are used to calculate impedance—comprised of resistance (R_rs_), a measure of airway calibre; and reactance (X_rs_) representing the elastic (compliance) components. Oscillometry has predominantly been used in obstructive respiratory diseases with a paucity of studies in patients with ILD. Studies have demonstrated increased R_rs_ and decreased X_rs_ in those with ILD [15, 16] compared to healthy controls [17] and people with mild-moderate COPD [18]. Conversely, other studies have demonstrated that R_rs_ in ILD, specifically interstitial pulmonary fibrosis, is normal yet X_rs_ is decreased [19, 20], likely reflecting reduced lung compliance from lung fibrosis [19]. Despite its increasing use in tertiary centers, including six in Sydney thus far, studies assessing oscillometry measurements post-LTx remain limited. One study identified physiological changes, increased R_5–19_ and reactance area (A_x_) and decreased X_5_, in biopsy-proven acute cellular rejection post-DLTx that were undetectable by spirometry [21]. Mathematical models have also been used to calculate impedance using various airway and lung tissue models to describe respiratory mechanics in different disease states [22]. However, none has examined oscillometry measurements in patients with ILD following LTx. Thus, combining our existing knowledge of oscillometry in other disease states and the lack of understanding in our study’s patient population, oscillometry may provide further useful pathophysiological insights in patients with ILD following LTx.

We hypothesized that in patients with ILD who have undergone SLTx, resistance (R_rs_) would be increased, reactance (X_rs_) decreased, and A_x_ increased compared to those post-DLTx. Thus, the aim of this study was to characterize resistance (R_5_ and R_5–19_) and reactance (X_5_) and A_x_ in stable recipients and evaluate the relationship between spirometry and oscillometry results following SLTx and DLTx for ILD.

## Materials and Methods

A cross-sectional study of adult LTx recipients performed for patients with ILD was undertaken at two Australia centers (Sydney and Melbourne), between January-2020 and May-2021. Patients attending routine clinic appointments were approached and consented to participate in the study. The study was initiated just prior to the COVID-19 pandemic which limited data collection. ILD was defined by a consensus clinical, physiological and radiological diagnosis. Donor and recipient matching and surgical techniques were performed as per standard clinical practice [23, 24]. Patients underwent unilateral or bilateral thoracotomy for SLTx and DLTx, respectively. For ILD recipients, lung donors for DLTx are selected based on the predicted total lung capacity (TLC), usually being between the recipients actual measured TLC and their predicted TLC. Lung donors for SLTx are typically larger than that of the recipients (i.e., oversized).

**FIGURE 1:**
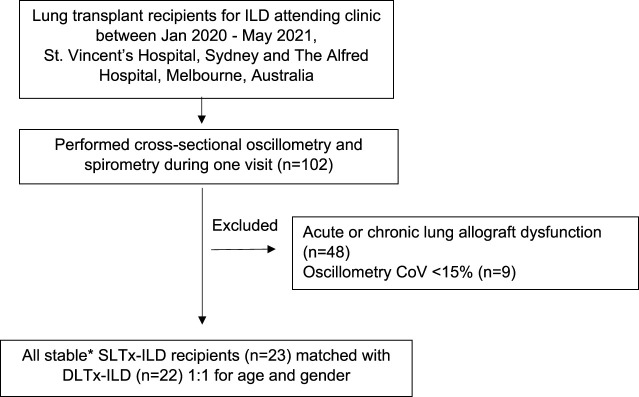
Flow diagram of inclusion and exclusion criteria for participant selection. Definition of abbreviations: ILD, Interstitial Lung Disease; CoV, Coefficient of variation; SLTx-ILD, Single lung transplant for ILD; DLTx, Double lung transplant for ILD. *Defined as concurrent/baseline FEV_1_ ≥ 90%.

LTx recipients with stable allograft function, defined as concurrent/baseline FEV_1_ ≥ 90%, were eligible for study enrolment [25]. Baseline FEV1 was defined as the best FEV_1_ measurement achieved post LTx. Recipients with acute or chronic lung allograft dysfunction were excluded [25] therefore bronchoscopy and transbronchial biopsy data were not included. Selected patient data were also used in Darley et al.’s recent study “Airway oscillometry parameters in baseline lung allograft dysfunction: Associations from a multicenter study,” whose results have no implications on this study [26]. Study participants performed oscillometry followed by spirometry during a single visit (Figure 1). Participants were classified into two groups (SLTx and DLTx) and were matched 1:1 for age and gender. Chest radiographs performed as part of standard clinical care within at least 6 months of the study visit were used as a surrogate measure of lung volumes in the SLTx group.

### Lung Function

Oscillometry measurements were performed using the tremoflo device (THORASYS^®^ tremoflo^®^ C-100 Airway Oscillometry System) according to European Respiratory taskforce recommendations [27]. Artefacts and tests that did not meet quality control (three measurements per patient with a R_5_ coefficient variation of <15%) were excluded [28]. Spirometry (Vmax Software, BreezeSuite) was performed as per American Thoracic Society/European Respiratory Society task force recommendations [29]. Standard oscillometry (R_5_, R_5–19_, X_5_, A_X_) and spirometry (FEV_1_, FVC, FEV_1_/FVC) parameters were reported. Z-scores for oscillometry and %-predicted values for spirometry measurements were calculated using published predictive equations [14, 30]. A normal Z-score was determined by ±one standard deviation from the mean (Z-score of ±1.64).

### Chest Radiographs

Digital chest radiograph (CXR) measurements [lung height and width (cm)]) were obtained from the allograft and native lung in the SLTx recipients. CXR measurements were performed using in-software Cerner Enterprise Web Viewer 3.0 calipers. Lung height was measured from the mid-diaphragm to the lung apex and width was measured from the inside of the chest wall across the mid-height of the two diaphragms [31].

### Modelling

Oscillometry measurements from patients with ILD and from the DLTx group were used in a standard model of respiratory impedance. ILD patients with an FVC measurement of <80% to match spirometry of the LTx groups were included. Patients with ILD (*n* = 25, male = 19) had a mean ± SD age of 72.2 ± 6.5 years and %-predicted FVC of 63.9% ± 10.6%.

**FIGURE 2:**
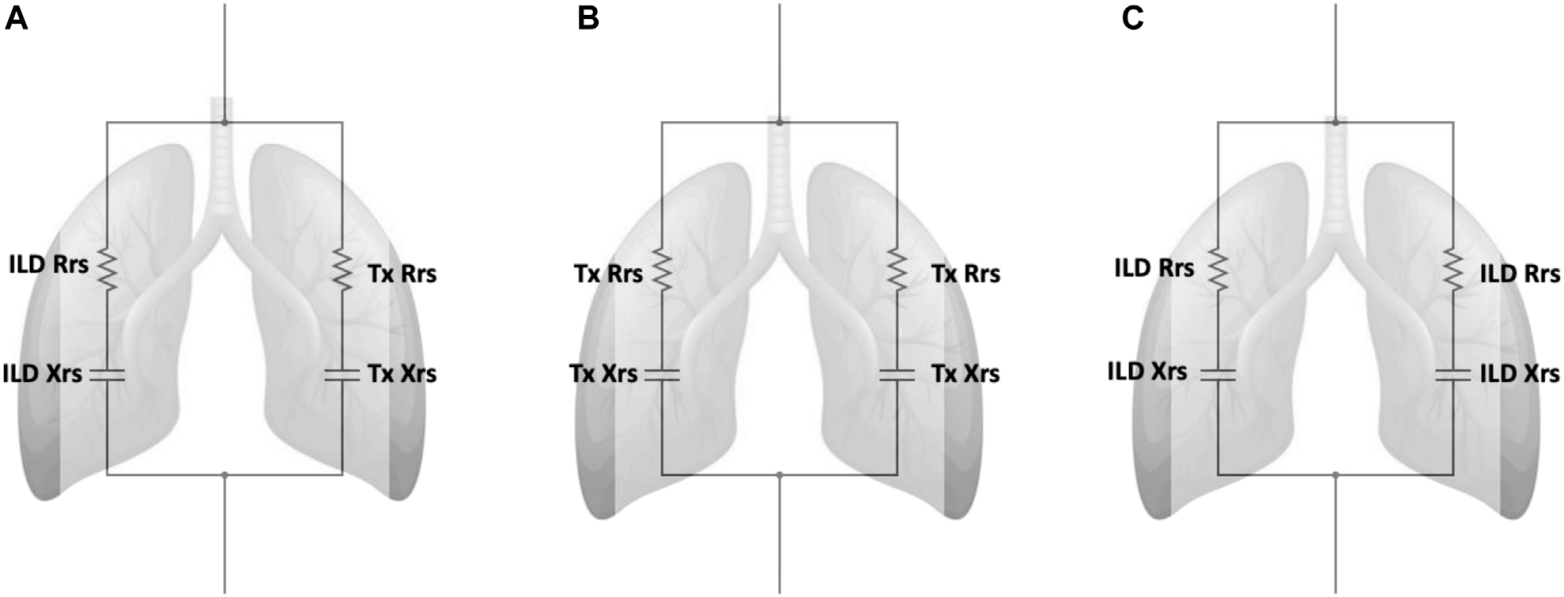
Inhomogeneous models with separate parallel pathways for each lung. Definition of abbreviations: ILD, Interstitial lung disease; Tx, Transplant; Rrs, Respiratory Resistance; Xrs, Respiratory Reactance. **(A)** Model of the single lung transplant group. This model contains separate parallel pathways with a single ILD lung and a single transplanted lung. **(B)** Model of the double lung transplant group. This model contains two parallel pathways of a single transplanted lungs. **(C)** Model of ILD group. This model contains two parallel pathways of a single ILD lungs. ILD Rrs and Tx Rrs are the mean resistance from a single lung from the ILD and Double lung transplant groups respectively. ILD Xrs and Tx Xrs are the mean reactance from a single lung from the ILD and Double lung transplant groups respectively.

In brief, the standard model obtained from oscillometry is typically expressed with separate resistive (*R*) and reactive (*X*) components (Figure 2). This model can be advanced to an inhomogeneous airway model with two parallel pathways (one for each lung) to examine resistance (R_rs_) and reactance (X_rs_) from each lung independently [32]. The model was used to determine the R_rs_ and X_rs_ contribution from a single lung in both the DLTx and ILD groups by using the median R_5_ and X_5_ from each group (Supplementary Equations S1, S2). Modelling of R_5_ and X_5_ for a SLTx recipient was derived by combining the results from a single lung from each of the DLTx and ILD groups (Figure 2). Further details are outlined in the Supplementary Material.

### Statistical Analysis

Statistical analyses were performed using GraphPad Prism 8.4.2 and IBM SPSS Statistics 26. Descriptive statistics were summarized using mean with standard deviation or median with interquartile range for continuous variables for parametric and non-parametrically distributed data, respectively; and frequency (%) for categorical variables. Results were compared using the two-sample *t*-test for continuous variables and the chi-square test for categorical variables. Relationships between oscillometry and spirometry were assessed using Spearman’s correlation. Statistical significance was set at a 2-sided level of 0.05.

## Guidelines

The study was approved by the St Vincent’s Hospital Human Research Ethics Committee (2019/ETH12765) and the Alfred Health Human Research Ethics Committee (HREC 50035).

## Results

A total of 45 stable recipients after LTx for ILD were recruited (23 SLTx and 22 DLTx recipients). Baseline demographics (Table 1) between the SLTx and DLTx groups were similar with regards to recipient gender (87.0% versus 77.3% males) and recipient and donor age [median (IQR) age for recipients: 63.0 (57.0–67.0) versus 63.0 (58.0–66.3) years and mean ± SD age for donors: 44.6 ± 12.9 versus 49.9 ± 18.1 years, for SLTx and DLTx, respectively]. Recipient height, weight and BMI, donor-recipient height difference and donor smoking history were similar between SLTx and DLTx groups. Concurrent FEV_1_/baseline FEV_1_% were also similar between the two groups [median (IQR) 96.0 (92.5–101.0)% versus 98.3 (94.5–100.0)% for SLTx and DLTx, respectively], indicating lung function stability and no evidence of chronic allograft dysfunction. The duration post-LTx was significantly shorter in the SLTx compared to the DLTx group [median (IQR) 1.0 (0.7–1.9) versus 1.6 (1.0–2.7) years (*p < 0.05*), for SLTx and DLTx, respectively]. Donor height was significantly taller in the SLTx compared to the DLTx group (mean ± SD 176.0 ± 6.7 versus 167.0 ± 11.0 cm) (*p < 0.01*). CXR measurements in the SLTx group demonstrated smaller height (169.2 ± 26.9 cm) and width (89.3 ± 13.0 cm) in the native lung compared to the allograft (207.0 ± 31.4 cm and 127.0 ± 22.0 cm, for height and width, respectively) (*p < 0.01*). Most CXRs (18/23 patients) were performed on the same day or within a month of lung function measurements. Three patients in the SLTx group had bronchial complications—two with left bronchial stenoses requiring stent insertion at four and 6 months prior to lung function measurements. One patient had a left anastomotic stricture.

**TABLE 1:** Baseline recipient and donor demographics of single and double lung transplant groups.

Patient characteristics	SLTx (*n* = 23)	DLTx (*n* = 22)	*p*-value
Recipient age (years)	63.0 (57.0–67.0)*	63.0 (58.0–66.3)*	0.78
Recipient height (cm)	172.0 (10.6)	171.0 (8.2)	0.60
Recipient weight (kg)	80.3 (12.7)	77.0 (15.7)	0.44
Recipient BMI (kg/m^2^)	26.9 (3.9)	26.5 (5.1)	0.61
Gender (*n*, % total)			
Males	20 (87.0%)	17 (77.3%)	0.46
Females	3 (13.0%)	5 (22.7%)	
Duration Post-transplant (years)	1.0 (0.7–1.9)*	1.6 (1.0–2.7)*	<0.05
Allograft side			
Left	8	22	-
Right	14	22	-
Types of ILD			
Idiopathic pulmonary fibrosis	14	18	-
Hypersensitivity pneumonitis	5	1	-
Connective tissue disease-ILD	1	0	-
Combined pulmonary fibrosis emphysema	1	1	-
Nonspecific interstitial pneumonia	1	1	-
Lymphoid interstitial pneumonia	1	0	-
Niemann-pick type B	0	1	-
Donor age (years)	44.6 (12.9)	49.9 (18.1)	0.28
Donor height (cm)	176.0 (6.7)	167.0 (11.0)	<0.01
Donor-recipient height difference (cm)	5.9 (4.0)	8.0 (5.4)	0.17
Donor smoking history (*n*, % total)			
No	9 (39.1%)	14 (63.6%)	0.20
Yes	11 (47.8%)	6 (27.3%)	
Not reported	3 (13.0%)	2 (9.1%)	
Spirometry post-LTx			
Concurrent FEV_1_/baseline FEV_1_ (%)	96.0 (92.5–101.0)*	98.3 (94.5–100.0)*	0.45
FEV_1_-% predicted	70.0 (14.5)	93.5 (26.0)	<0.01
FVC-% predicted	70.5 (16.8)	90.7 (12.8)	<0.01
Concurrent FEV_1_/FVC	0.80 (0.098)	0.80 (0.080)	0.81

All data are reported as mean (SD) or median (IQR)*. Definition of abbreviations: SLTx, Single Lung Transplant; DLTx, Double Lung Transplant; BMI, body mass index; FEV_1_, forced expiratory volume in one second; FVC, forced vital capacity.

### Lung Function

FEV_1_ and FVC were significantly lower in the SLTx group compared to the DLTx group (Table 1). Mean ± SD FEV_1_-% predicted was 70.0 ± 14.5 versus 93.5% ± 26.0% (*p < 0.01*) and FVC-% predicted was 70.5 ± 16.8 versus 90.7% ± 12.8% (*p < 0.01*), in SLTx and DLTx groups, respectively. Oscillometry demonstrated that R_5_ in both SLTx and DLTx groups were within normal limits (median Z-score <1.64). However, X_5_ and A_x_ were abnormal in the SLTx group (median Z-scores of −2.26 and 2.22 for X_5_ and A_x_, respectively) and within normal limits in the DLTx group (Table 2).

**TABLE 2:** Oscillometry data in the single and double lung transplant groups and ILD group.

	SLTx *n* = 23	DLTx *n* = 22	*p*-value	ILD *n* = 25	Model data (single lung)
R_5_ (cmH_2_O.s/L)	3.06 (2.67–3.83)	3.06 (2.48–3.84)	0.94	3.41 (2.85–3.69)	3.23
Z-score	0.61 (−0.18 to 1.29)	0.11 (−0.79 to 1.27)	0.54	0.002 (−0.60 to 1.29)	—
Z-score >1.64, *n*	3	4	—	4	—
R_5-19_ (cmH_2_O.s/L)	0.66 (0.45–1.08)	0.36 (0.08–0.78)	0.11	0.81 (0.63–1.20)	—
X_5_ (cmH_2_O.s/L)	−1.88 (−2.89 to −1.39)	−1.22 (−1.87 to −0.86)	<0.01	−2.24 (−2.74 to −1.97)	−1.73
Z-score	−2.26 (−3.76 to −0.83)	−0.36 (−1.44 to 0.37)	<0.01	−2.52 (−3.53 to −1.42)	—
Z-score <−1.64, *n*	14	4	—	16	—
A_x_ (cmH_2_O/L)	13.00 (9.73–18.50)	7.58 (3.55–13.50)	0.01	17.0 (13.65–22.22)	—
Z-score	2.22 (1.52–2.68)	1.17 (0.44–2.25)	0.01	2.21 (1.62–2.68)	—
Z-score >1.64, *n*	17	9	—	19	—

All data are reported as median (IQR)* Definition of abbreviations: SLTx, Single Lung Transplant; DLTx, Double Lung Transplant; ILD, interstitial lung disease; R_5_, resistance at 5 Hz; R_5–19_, Resistance between 5 and 19 Hz; X_5_, Reactance at 5 Hz; A_x_, Reactance Area.

**FIGURE 3:**
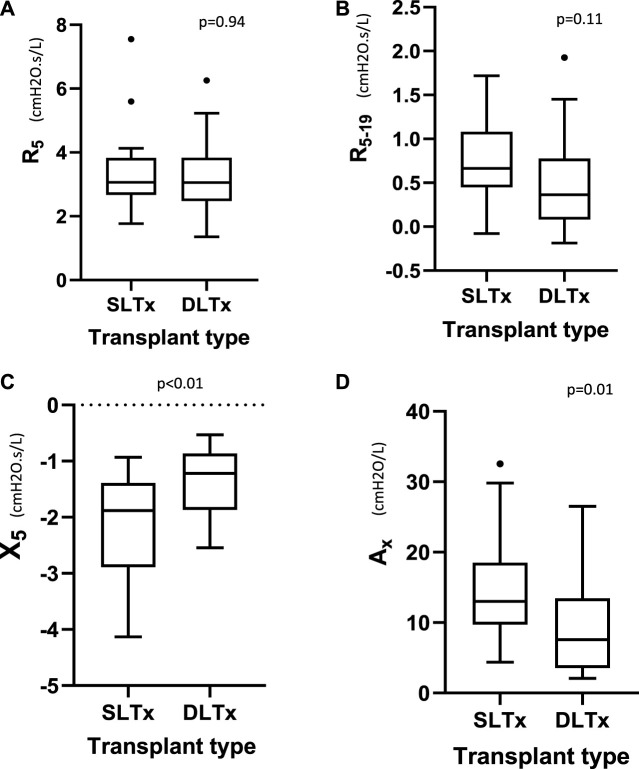
Tukey boxplot comparing the oscillometry indices of **(A)** R_5_, **(B)** R_5-19_, **(C)** X_5_, **(D)** A_x_ in 23 SLTx and 22 DLTx patients. Outliers are marked with dots outside the boxplots. Definition of abbreviations: SLTx, Single Lung Transplant; DLTx, Double Lung Transplant; R_5_, resistance at 5Hz; R_5-19_, Resistance between 5Hz and 19Hz; X_5_, Reactance at 5Hz; A_x_, Reactance Area.

Oscillometry showed similar measurements in resistance (R_5_ and R_5–19_) between both groups. Median (IQR) R_5_ was 3.06 (2.67–3.83) versus 3.06 (2.48–3.84) cmH_2_O.s/L (*p = 0.94*) and R_5-19_ was 0.66 (0.45–1.08) versus 0.36 (0.08–0.74) cmH_2_O.s/L (*p = 0.11*) in the SLTx and DLTx groups, respectively. Reactance (X_5_) was significantly lower and A_x_ significantly higher (i.e., more abnormal) in the SLTx group compared to the DLTx group. Median (IQR) X_5_ was −1.88 (−2.89 to −1.39) versus −1.22 (−1.87 to −0.86) cmH_2_O.s/L (*p < 0.01*) and A_x_ was 13.00 (9.73–18.50) versus 7.58 (3.55–13.50) cmH_2_O/L (*p = 0.01*) in the SLTx and DLTx groups, respectively (Table 2; Figure 3). Z-score comparisons of oscillometry measurements between SLTx and DLTx groups were similar to that observed with raw values (Table 2).

There were significant associations between oscillometry parameters (R_5_, R_5–19_, X_5_ and Ax) and FVC in the SLTx group [R_5_ (rs = −0.47, *p* = 0.02), R_5-19_ (rs = −0.45, *p* = 0.03), X_5_ (rs = 0.72, *p* < 0.01) and Ax (rs = −0.70, *p* < 0.01)]. In the DLTx group, significant correlations with FVC were only demonstrated between X_5_ (rs = 0.65, *p* < 0.01) and Ax (rs = −0.52, *p* = 0.01). Similar correlations were observed when comparing FEV_1_ with oscillometry indices for both SLTx and DLTx groups.

### Modelling

The derived single lung values of R_5_ and X_5_ for DLTx and ILD groups are displayed in Table 2. There was close agreement between the inhomogeneous oscillometry model predicted R_5_ (3.23 cmH_2_O.s/L) and X_5_ (−1.73 cmH_2_O.s/L) with the measured R_5_ (3.06 cmH_2_O.s/L) and X_5_ (−1.88 cmH_2_O.s/L) in the SLTx group.

## Discussion

Our multicenter cross-sectional study is the first study, to our knowledge, to report oscillometry measurements in stable single (SLTx) and double (DLTx) lung transplantation recipients, exclusively in patients with ILD as their native lung disease. Our novel findings demonstrate that resistance (R_5_ and R_5–19_) measured by oscillometry was similar between SLTx and DTLx recipients despite FEV_1_ and FVC being significantly lower in the SLTx group. Furthermore, reactance at 5 Hz (X_5_) and A_x_ were significantly worse in the SLTx recipients compared to the DLTx recipients. These findings were replicated using a simple mathematical model based on real-life data obtained from DLTx recipients and patients with ILD. Our data, suggests that the differences in respiratory mechanics after SLTx and DLTx may be predominantly attributed to changes in the elastic properties rather than airway caliber.

Resistance (R_5_ and R_5–19_) was not increased (i.e., not more abnormal) in the SLTx compared to DLTx recipients. This may be due to patients in our study having stable disease as indicated by the preserved spirometric ratio and concurrent/baseline FEV_1_ being greater than 90% [25] and thus suggesting the absence of spirometric obstruction and acute or chronic lung allograft dysfunction. Chronic allograft dysfunction is commonly due to bronchiolitis obliterans (BO) [33] with the underlying pathology being fibroproliferative airway plugging [34]. Airway plugging may lead to a reduction in airway caliber and an increase in airway resistance. As resistance was similar between SLTx and DLTx recipients, allograft dysfunction due to BO seems unlikely. This is supported by our cohort being spirometrically-stable. The underlying pathology in the native single ILD lung typically affects the lung parenchyma rather than the airways. However, airway epithelial cell proliferation and expansion in a number of bronchioles can also occur in the distal airways of those with ILD [35]. We speculate that changes in the distal airways may increase airway caliber in the native single ILD lung and thus explain the similarities in resistance between the SLTx and DLTx recipients. Our results are consistent with recent oscillometry studies demonstrating normal resistance in ILD [19, 20]. However, data is conflicting as other studies report resistance to be increased or impaired in patients with ILD in those with more severe lung restriction and lung function impairment [17]. Comparatively, in our study, spirometry demonstrated that lung function impairment was worse in our SLTx recipients compared to DLTx recipients, yet resistance derived from oscillometry was not. Comparisons with other studies are limited because previous oscillometry studies examined ILD patients that did not include LTx recipients.

In contrast to resistance, reactance (X_5_) was significantly lower, and A_x_ was significantly higher (i.e., X_5_ and A_x_ were more impaired) in the SLTx compared to the DLTx recipients. These findings are consistent with previous studies showing more abnormal reactance in patients with ILD compared to healthy controls [17, 20] and in those with ILD and more severe lung restriction [15]. Reduced lung volume due to the diseased native ILD lung could account for X_5_ and A_x_ being more abnormal as these parameters are dependent on lung volume [36]. In the SLTx recipients the native ILD lung was significantly smaller compared to the allograft, which we confirmed using chest radiograph measurements. The allograft side may have contributed to lung volume differences in the SLTx group because left-sided allografts are typically smaller because of the position of the heart. However, a majority of our SLTx recipients underwent a right-sided LTx thus unlikely to contribute to our results (Table 1). Differences in lung volumes between the native lung and allograft in SLTx recipients may lead to asynchrony and altered lung mechanics during respiration. This phenomenon has not been demonstrated in SLTx recipients with ILD, but asynchrony can occur in SLTx recipients with emphysema. The native emphysematous lung and allograft can inflate and empty at different rates and subsequently lead to chest wall asymmetry and mediastinal shift during respiration [12]. The reduced lung volumes may therefore explain a more abnormal reactance. The forced maneuver during spirometry versus tidal breathing during oscillometry measurement needs to be taken into consideration, however the impact on the resulting physiological measurements remains elusive. Additionally, asynchrony in muscle forces, which may result from diaphragm dysfunction, can develop between the two sides of the chest after SLTx [37] and may exacerbate chest wall asymmetry and alter chest wall and lung mechanics. Studies assessing reactance measured via oscillometry in patients with SLTx, respiratory muscle dysfunction and/or chest wall deformities are lacking therefore we can only speculate these mechanisms.

Our study included a simple model that incorporated measurements from real-life ILD and LTx patients to support our *in vivo* findings in SLTx recipients. The inhomogeneous model shows that in the SLTx group, the single transplanted lung has low reactance while the non-transplanted lung has high reactance (i.e., an increased X_5_) which corroborates our novel findings. Agreement between the predicted X_5_ from the model and the measured X_5_ in the actual SLTx group further ascertains that the increased X_5_ measured in the SLTx group is indeed attributed to the increased reactance in the native ILD lung. While there is close agreement between the predicted and measured median X_5_, the measured X_5_ was slightly more abnormal (−1.73 versus −1.88 cmH_2_O.s/L, respectively). The more negative X_5_ may be a reflection of more advanced disease in the SLTx group before transplantation. Using the ILD group’s single lung reactance in the SLTx model, we may have underestimated the reactance in the single native ILD lung. The results derived from this model replicates and provides further evidence to support our *in vivo* findings in a small number of ILD patients after single and double lung transplantation.

Spirometry was significantly lower or more impaired in our SLTx recipients compared to DLTx recipients as demonstrated in other studies [9, 10]. Anthropometrics in the SLTx and DLTx recipients were similar and thus unlikely to contribute to differences in spirometry. Donor height was significantly taller in the SLTx recipients however is unlikely to be relevant because there was no difference in donor-recipient height matching between the two groups, suggesting appropriate lung size matching. The maximal spirometry measurement achieved is typically lower in SLTx compared to DLTx recipients [10, 38] and thought to be related to the remaining single diseased native lung. The disease pathology in the native lung is also reflected in the normal FEV_1_/FVC ratio in SLTx, consistent with that of restrictive lung disease.

Limitations that must be acknowledged include the small sample size in our study. The patient cohort was small, as we only included patients with ILD as their native disease. Only one other study has measured oscillometry in SLTx and DLTx recipients however these authors assessed LTx recipients with various forms of native lung diseases, with COPD comprising the majority of their patient cohort [39]. Limiting our study participants to one native disease, ILD, avoids confounding factors from including various diseases. Furthermore, our study groups were matched for age and gender and there were no significant differences between recipient baseline characteristics to confound our results. Differences in lung volume likely contribute to our findings and additionally we did not report lung volume measurements. As a surrogate we showed that there was a significant difference in lung size between the native and allograft lung in the SLTx group using a standardized technique of chest radiograph measurements [31]. The effect of significant differences between donor and recipient height must also be acknowledged however, optimum size matching was performed in accordance with local guidelines. There was no significant difference in smoking history between the two groups and the effect of donor smoking is not known but donor smoking history must also be acknowledged.

The time post-LTx was statistically significantly shorter in the SLTx compared to the DLTx group, however it is clinically insignificant since both groups should have achieved and maintained their maximal spirometry at the time of measurement during the study [9]. The specific effect of relevant clinical parameters such as bronchial stenosis and/or other bronchial or pleural complications were not examined in this cross-sectional study and require further evaluation. Furthermore, the trajectory of oscillometry measurements is not established and will likely alter over time. Spirometry declines more rapidly in SLTx than in DLTx recipients [9, 40] and whether this also occurs in oscillometry is yet to be determined.

## Conclusion

In summary, in SLTx recipients, oscillometry measurement of resistance is similar to that observed in DLTx recipients. However, similarly to spirometry, reactance is more impaired in SLTx compared to DLTx recipients. This is likely attributed to changes in the elastance due to reduced alveolar volume in the native ILD lung in SLTx recipients and may lead to asynchrony in respiratory mechanics. Whether the breathing maneuver performing during lung function testing impacts respiratory mechanics is yet to be elucidated but “quiet” tidal breathing may be a more attractive measurement compared to the forced maneuver used in spirometry.

These cross-sectional findings highlight the physiological complexities of LTx that are not completely understood. The significance of normal resistance, yet abnormal spirometry and abnormal reactance as a predictor of clinical outcomes, requires reliable reference values and further longitudinal investigation. Further study in LTx recipients with obstructive lung disease would also improve our understanding. A better understanding of the physiological changes after SLTx and DLTx is vital for developing novel diagnostic and therapeutic approaches to improve LTx outcomes.

## Data Availability

The original contributions presented in the study are included in the article/Supplementary Material, further inquiries can be directed to the corresponding author.

## Abbreviations

A_x_, Reactance Area; BO, Bronchiolitis obliterans; DLTx, Double Lung Transplantation; FEV_1_, Forced Expiratory Volume in one second; FVC, Forced Vital Capacity; ILD, Interstitial Lung Disease; R_5_, Resistance at 5 Hz; R_5–19_, Resistance between 5 Hz and 19 Hz; SLTx, Single Lung Transplantation; TLC, Total Lung Capacity; X_5_, Reactance at 5 Hz.
