# Supplementary material for: Oscillometry in Stable Single and Double Lung Allograft Recipients Transplanted for Interstitial Lung Disease: Results of a Multi-Center Australian Study
Source: Transpl Int. 2023 Dec 5;36:11758. doi: 10.3389/ti.2023.11758 (PMC10728296; doi:10.3389/ti.2023.11758)
Supplement: Supplementary file 1 [file DataSheet1.docx]

Online Supplement

**Estimation of resistance and reactance for single lungs**

Total resistance is calculated from parrel resistive components (equation 1), as below.

$Rrs\left( total \right)=\frac{1}{Rrs(left lung)}+\frac{1}{Rrs(right lung)}$ (equation 1)

Where Rrs(total) is the total respiratory resistance, Rrs(left lung) is the resistance of the left lung and Rrs(right lung) is the resistance of the right lung.

Total reactance is calculated from parallel capacitive components (equation 2), as below.

$Xrs\left( total \right)=Xrs(left lung)+Xrs(right lung)$ (equation 2)

Where Xrs(total) is the total respiratory reactance, Xrs(left lung) is the reactance of the left lung and Xrs(right lung) is the reactance of the right lung.

|  | **ILD** | **DLTx** | **SLTx** |
| --- | --- | --- | --- |
| Rrs(total) | $=\frac{1}{Rrs(ILD)}+\frac{1}{Rrs(ILD)}$ | $=\frac{1}{Rrs(Tx)}+\frac{1}{Rrs(Tx)}$ | $=\frac{1}{Rrs(ILD)}+\frac{1}{Rrs(Tx)}$ |
| Xrs(total) | $=Xrs(ILD)+Xrs(ILD)$ | $=Xrs(Tx)+Xrs(Tx)$ | $=Xrs(Tx)+Xrs(ILD)$ |

Table S1: Mathematical description of the inhomogeneous models used. *Definition of abbreviations:* ILD=Interstitial lung disease; DLTx=Double Lung Transplant; SLTx=Single Lung Transplant, Rrs=Respiratory Resistance, Tx=Transplant, Xrs=Respiratory Reactance

Where Rrs(ILD) and Rrs(Tx) are the mean resistance from a single lung from the ILD and Double lung transplant groups, respectively. Xrs(ILD) and Xrs(Tx) are the mean reactance from a single lung from the ILD and Double lung transplant groups, respectively.

The mean R_5_ and X_5_ measured from the ILD, and Double lung Tx groups were used as the total resistance (Rrs) and reactance (Xrs) values and used to calculate the mean single lung values for each group (equation 1 and equation 2). The modelled single lung values from the ILD (Rrs(ILD) and Xrs(ILD)) and double Tx groups (Rrs(Tx) and Xrs(Tx)) were used to predict R_5_ and X_5_ in the single lung transplant group (Table 2, Figure 2). Predicted values from the model were compared to measured values in the single lung transplant group.
